# Stool Phospholipid Signature is Altered by Diet and Tumors

**DOI:** 10.1371/journal.pone.0114352

**Published:** 2014-12-03

**Authors:** Julie M. Davies, Hong-Uyen Hua, Rishu Dheer, Mitchell Martinez, Sanjoy K. Bhattacharya, Maria T. Abreu

**Affiliations:** 1 Division of Gastroenterology, Department of Medicine, Miller School of Medicine, University of Miami, Miami, Florida, United States of America; 2 Bascom Palmer Eye Institute, University of Miami, Miami, Florida, United States of America; University of Palermo, Italy

## Abstract

Intake of saturated fat is a risk factor for ulcerative colitis (UC) and colon cancer. Changes in the microbiota have been implicated in the development of UC and colon cancer. The host and the microbiota generate metabolites that may contribute to or reflect disease pathogenesis. We used lipid class specific quantitative mass spectrometry to assess the phospholipid (PL) profile (phosphatidylcholine [PC], phosphatidylethanolamine [PE], phosphatidylinositol [PI], phosphatidylserine [PS]) of stool from mice fed a high fat (HFD) or control diet with or without induction of colitis-associated tumors using azoxymethane and dextran sodium sulfate. The microbiota was assessed using qPCR for several bacterial groups. Colitis-associated tumors were associated with reduced bulk PI and PE levels in control diet fed mice compared to untreated mice. Significant decreases in the relative quantities of several PC species were found in colitis-associated tumor bearing mice fed either diet. Statistical analysis of the PL profile revealed distinct clustering by treatment group. Partial least squares regression analysis found that the relative quantities of the PS class profile best predicted bacterial abundance of *Clostridium leptum* and *Prevotella* groups. Abundance of selected PL species correlated with bacterial group quantities. Thus, we have described that a HFD and colitis-associated tumors are associated with changes in phospholipids and may reflect host-microbial interactions and disease states.

## Introduction

Obesity and a high intake of saturated fats are risk factors for ulcerative colitis and colon cancer [1]–[5]. The extent and duration of inflammation in ulcerative colitis patients is highly predictive of cancer development and 18–30% of UC patients with extensive colitis will develop colorectal dysplasia or cancer [6], [7] within thirty years. Age of onset between 20–39 years also increases the relative risk of developing cancer after 20 years [8]. In the intestine, a high fat diet (HFD) has been shown to increase epithelial permeability [9], colonic inflammatory markers [10], [11], and exacerbate dextran sodium sulfate (DSS)-induced colitis [12]. Therefore, obesity and a HFD have systemic effects, but also have profound effects on the local intestinal environment.

This study focuses on the effect of diet and neoplasia on phospholipids (PLs) found in the colonic lumen. Phospholipids are the major component of cell membranes and are also important intracellular signaling molecules. Phospholipids contain a polar head group and two hydrocarbon tails which add enormous diversity to their structure and possibly also their function. Dietary lipids are 90% triglycerides and 10% other lipids including: cholesterol esters, plant sterols and PLs. Phospholipids are hydrolyzed in the small intestine by PLA_2_ and absorbed by enterocytes and delivered to lymph or directly enter the portal blood depending on chain length [13], indicating that dietary intake does not greatly contribute to the stool PL pool. Phospholipids in the stool are derived from three main sources: bile (mainly PC), shed epithelial cells, and bacterial cells. Stool is a readily available resource for investigating colonic function, and isolation of lipids from stool has recently been validated by Gregory *et al.* from fecal matter of premature infants and LC/MS for lipid species analysis [14].

The microbiota is a critical component of the intestinal environment and is altered by changes to diet and obesity [15]. An increase in Firmicutes and a decrease in Bacteroides have been observed in both mouse and human obesity studies [16]–[18]. Transfer of microbiota from genetically obese mice to lean mice increases weight gain indicating that the microbiota plays a dominant role in energy extraction [18]. The microbiota rapidly alters in response to changes in diet—within 24 hours changes to the microbiota are detectable [19]. However, over the long-term, microbial populations are generally stable. Given the role of the microbiota in metabolism, examining the interplay between the microbiota and biologically-relevant metabolites in inflammation-associated dysplasia may elucidate biochemical pathways and biomarkers to improve human disease.

Elegant work has pioneered the analysis of the interaction between the microbiota and metabolism [20]–[22] – alternately named “metabonomics”. Findings from these studies have demonstrated that microbiota-dependent metabolic differences occurring between conventional and germ-free mice are measurable not only locally in colonic epithelial cells, but also systemically in urine, kidney and liver [20], [21], [23]. Metabolic adaptations of colon cancer samples have identified profiles of metabolites including amino acids, monosaccharides and fatty acids that track with disease [24], [25]. However, the phospholipid profile in these studies was not examined.

To determine how colitis-associated tumor development under different dietary conditions alters the stool PL profile we fed mice either a control diet (10% calories from fat) or HFD (60% calories from fat) and colitis-associated tumors were induced with a standard protocol [26]. Our results demonstrate that the stool lipid profile was altered by: changes in diet, the presence of tumors, and tumors occurring under different dietary conditions. Additional examination of the relative abundance of several stool bacterial groups allowed us to correlate relative PL quantities with bacterial group quantities. As stool PLs are derived from both host and microbiota their measurement examines the interplay between both systems. To our knowledge this is the first study wherein a targeted lipidomic approach has identified the phospholipid profile in stool obtained from a murine model of colitis-associated tumorigenesis.

## Methods

### Ethical Statement

All studies using mice were approved by and performed according to the University of Miami Institutional Animal Care and Use Committees' (IACUC) guidelines (Protocol number 11-053). All efforts were made to minimize animal suffering, and animals were sacrificed at the end of the study by cervical dislocation.

### Study design

Eight week old male and female C57BL6 mice were randomized to four treatment groups consisting of four to six animals per group (control diet no Tx = 4 mice, HFD no Tx = 4, Ctl diet AOM-DSS = 4, HFD AOM-DSS = 6). The parental animals were obtained from Jackson laboratories (Bar Harbor, Maine) and experimental animals were bred in-house. Mice were maintained in ventilated cages in our SPF facility with a 12/12 hour light/dark cycle. Mice were fed either a control low fat diet (10% calories from fat (TD.06412) – Harlan, Indianapolis IN) or a HFD (60% calories from fat (TD.06414) – Harlan, Indianapolis IN) *ad libitum* for 2 weeks prior to commencement of tumor induction protocol. Tumors were induced by intraperitoneal injection of azoxymethane (AOM) (7.4mg/kg Sigma, St Louis MO). Two weeks later mice were given 2.5% dextran sodium sulfate (DSS) (MP Biomedicals, Solon OH) in their drinking water for 5 days. The DSS was removed for 9 days of recovery before another round of 2.5% DSS was administered for a further 5 days. Stool for lipid analysis was collected 2 weeks after the removal of the second round of DSS. Colons of mice were excised for visual tumor count.

**Figure 1:**
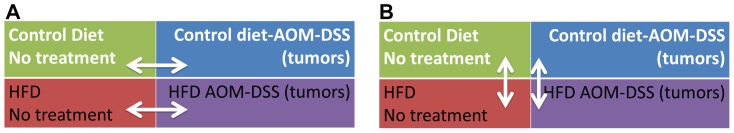
Schematic of analysis groups in study.

In most analyses we compared the impact of AOM-DSS induced tumors compared to untreated dietary controls (**Figure 1A**). However, in some instances we also compared the impact of the diet in untreated (control diet no treatment vs HFD no treatment) and in tumor-bearing mice (control diet AOM-DSS vs HFD AOM-DSS) (**Figure 1B**) on phospholipid compositions as outlined in the schematic.

**Figure 2:**
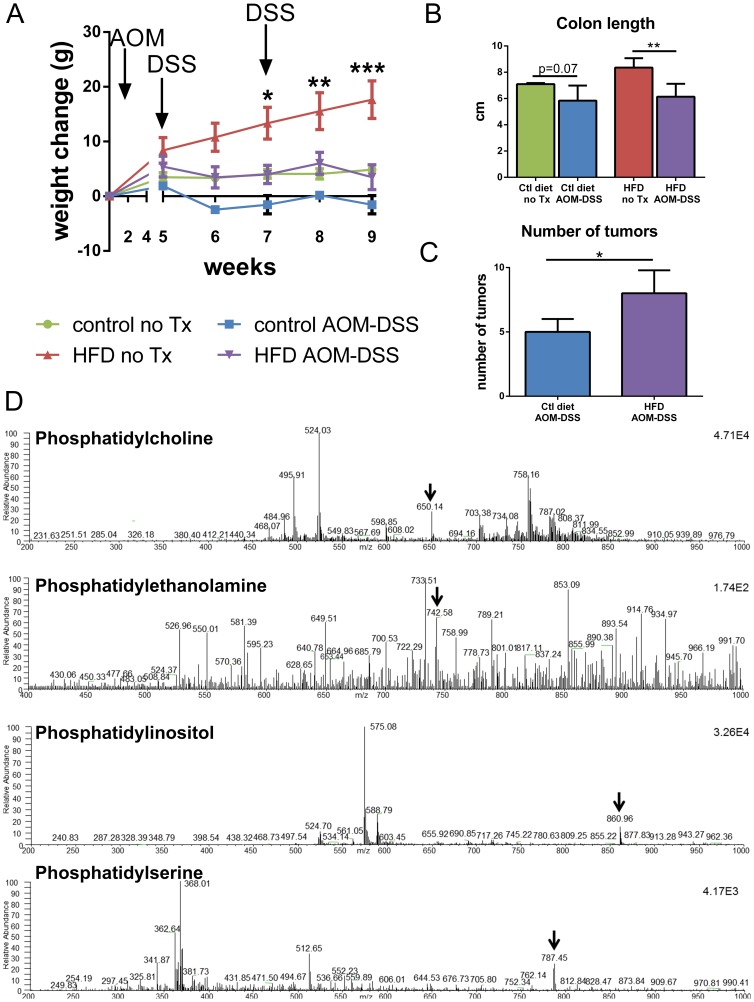
Outcome of feeding a HFD and tumor induction. **A**) Weight change over the course of the 10 week experiment. Weight change from the beginning of the experiment is plotted. Significance determined by Two-Way ANOVA compared to Ctl diet no Tx. Bonferroni post-hoc test. **B**) Length of excised colons was measured (cm). Bars represent means ±SD. Significance determined by unpaired t test compared to untreated dietary control. **C**) Tumors were visually counted in the colons of mice at the end of the treatment period. Bars represent means ±SD. Significance determined by unpaired t-test. Ctl diet AOM-DSS n = 4, HFD AOM-DSS n = 6. **D**) Representative electrospray ionization mass spectrometric analyses of phospholipid class. Arrows represents the internal standard. Precursor ion scan (PIS) for PC was conducted in positive ion mode with internal standard at 650.14 *m/z*. Precursor ion scan for PE was conducted in negative ion mode with internal standard at 742.58 *m/z*. Precursor ion scan for PI was conducted in negative ion mode with internal standard at 860.96 *m/z*. Representative neutral loss scan (NLS) for PS was conducted in negative ion mode with internal standard at 787.45 *m/z*. Significance demonstrated as ***p<0.001, **p<0.01, *p<0.05.

All individual mice given the tumor induction protocol developed polyps (100% penetrance). Multiplicity of tumor burden is visualized in **Figure 2B** **.**

### Lipid Extraction

Stool was processed by 5 repeated cycles of 5 minutes in liquid nitrogen followed by 5 minutes at 37°C in a water bath. Stool was then minced finely with scissors until powder. Lipids were extracted using the Bligh and Dryer method [27] with a few modifications. This method has been found to be superior for samples containing 5% lipids on a weight by weight basis over other methods for extraction of lipids from animal tissues [28]. After homogenization in chloroform: methanol (1∶1) for 2 minutes, additional chloroform was added (final 2.5∶1 chloroform:methanol), the sample was briefly centrifuged and the liquid removed from the insoluble sediment to a new tube. Additional water was added to fully allow the separation of the organic and aqueous phase. Samples were then centrifuged for 15 minutes at 14,000 g at 4°C. The organic bottom layer was transferred to a new tube and dried using a Speed-Vac (Model 7810014; Labconco, Kansas City, MO). Samples were flushed with argon gas prior to and after the drying process. Samples were kept at −80°C until analysis.

### Mass Spectrometric Analysis

Extracted lipids were resuspended in LC-MS grade acetonitrile:isopropanol (1∶1) and analyzed on a triple quadrupole electrospray mass spectrometer (TSQ Quantum Access Max; Thermo Fisher Scientific, Pittsburgh, PA). Lipid analysis was performed in infusion mode assisted by a Triversa Nanomate (Advion Inc., Ethaca, NY) using TSQ Tune software (Xcaliber 2.3). The nanomate was controlled using ChipSoft 8.3.3. Samples were analyzed for 2.00 min with a 0.500 s scan. Scans typically ranged from 200 *m*/*z* to 1000 *m*/*z* unless specified otherwise. Collision gas pressure was set at 1mTorr and analysis was carried out at 0.7 FWHM. Sheath gas (nitrogen) was set to 20 arbitrary units. Auxiliary gas (Argon) was set to 5 arbitrary units. For analyses of different phospholipid classes, collision energy, spray voltage, and ion mode were set based on previous studies [29]–[31]. Class specific lipids were quantified using class specific quantitative lipid standards in two steps [29]. In the first step the most abundant lipids of the class were quantified using a class specific lipid standard and in the second step, the quantification values determined using the first step were used for quantification of the identified low abundant lipid species [29]. The quantification lipid standards (Avanti Polar Lipids, Albaster, AL) were the same as in our previous study on trabecular meshwork, namely 1,2-ditridecanoyl-sn-glycero-3-phosphocholine (molecular mass 649.89, catalog no. 850340), 1,2-dioleoyl-sn-glycero-3-phospho-l-serine (molecular mass 810.03, catalog no. 840035), 1,2-dioleoyl-sn-glycero-3-phosphoethanolamine (molecular mass 744.04, catalog no. 850725) and 1,2-dioleoyl-sn-glycero-3-phospho-(10-myo-inositol) (molecular mass 880.15, catalog no. 850149) [30]. About 5 scans each with (0.1–2 pmol) and without internal standard was performed for each sample. Ratiometric quantification was achieved using the MZmine 2.9 [32] and/or Lipid Search program.

### Stool microbiota analysis

Genomic DNA was extracted from stool pellets using QIAamp stool DNA extraction kit (Qiagen, Valencia, CA) following manufacturer's instructions. Bacterial composition of the stool samples was assessed by automated ribosomal intergenic spacer analysis (ARISA) as described previously with some modifications [33]. Briefly, the intergenic regions between bacterial 16S and 23S rRNA genes were amplified using broad range primers 1406F (labeled at the 5′end with the phosphoramidite dye 5-FAM) and 23Sr. The intergenic lengths of 6-FAM labeled PCR products were determined on an ABI 3730 capillary sequencer (Applied biosystems, Grand Island, NY) using LIZ-1200 size standard and electropherograms were analyzed using peak scanner (Applied biosystems, Grand Island, NY). Real-time qPCR was performed on stool DNA using group-specific primers [34] to determine the relative abundance of individual bacterial groups. A standard curve for each primer pair was plotted using plasmid with appropriate 16S rRNA gene sequence insert to quantify the qPCR values into 16S copy number/g of normalized stool.

### Data analysis

Spectral peak areas were converted to pmol by comparing against the peak area of the internal standard (PC(13∶0/13∶0), PE(18∶1(9Z)/18∶1(9Z)), PI(18∶1(9Z)/18∶1(9Z)), PS(18∶1(9Z)/18∶1(9Z))) (Avanti Polar Lipids, Albaster, AL). Concentration was expressed per gram of stool or as a normalized ratio of stool weight between samples (“norm·g”). Mass/Charge (*m/z*) values displayed in tables are averages of all samples.

Bulk quantity of each PL class was defined as the sum of the pmol amount per gram of stool for both identified and unidentified species from the chromatograph of each lipid class.

Simpson's reciprocal index was calculated as 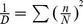
where *n* =  amount of each PL species and *N* = the total quantity of each class of PL.

Quantitative comparisons of the lipids species were assessed using the relative quantity of each lipid species compared to the total quantity in each class (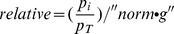) where p_i_ = pmol of the individual species and p_T_ = the total quantity of the PL class). Prior to analysis, PL species that were not present in at least half of the samples for one group were removed. Each class of phospholipid was assessed using a two-way ANOVA Bonferroni post-hoc test of common lipid species for quantitative differences occurring due to: diet (Ctl diet no Tx vs HFD no Tx), the presence of tumors (Ctl diet vs Ctl diet AOM-DSS and HFD no Tx vs HFD AOM-DSS) and tumors in different dietary environments (Ctl diet AOM-DSS vs HFD AOM-DSS).

Low abundance species were identified as species that occurred in less than half of the samples in one group. We compiled a list of all these species and removed those species that were not in present in at least half of the samples in any one of the other groups, thus removing species that were present in too few samples to analyze. The frequency occurrence of the lipid species was uploaded to CIMminer (Genomics and Bioinformatics Group, Laboratory of Molecular Pharmacology (LMP), Center for Cancer Research (CCR) National Cancer Institute (NCI)) to generate a heat map.

Raw data for this study can be found in the Excel file Table S3.

### Statistics

Partial least squares – discriminant analysis (PLS-DA) and Partial least squares regression analysis were conducted using Multibase Excel plug in (Numerical Dynamics). As with the Two-way ANOVAs, PLS-DA and partial least squares regression were performed on PL species that were found to be present in at least half of the samples in at least one group. For partial least squares regression analysis, relative quantities of PL species were used as the descriptor matrix (X) and the relative bacterial groups quantity used as the response variable (Y). One-way ANOVAs were performed with a Tukey post-hoc test. Two-way ANOVAs were performed with a Bonferroni correction. ANOVAs and Pearson correlation values were obtained using Graphpad Prism 6.02. Significance was annotated as follows: * = p<0.05, ** = p<0.01, *** = p<0.001, **** = p<0.00001.

## Results

### A HFD increases tumors in colitis-associated neoplasia

To investigate the impact of both a HFD and colitis-associated neoplasia on the stool phospholipid profile, mice were fed either a control diet (10% calories from fat) or a HFD (60% calories from fat) for two weeks prior to the tumor induction protocol. The fat source for both diets was lard and soybean oil. Mice fed the HFD gained significantly more weight than control diet fed mice over the experimental time frame. Administration of the tumor induction protocol in control diet and HFD fed mice decreased weight gain such that HFD fed mice were not obese at sacrifice (**Figure 2A**). Colon shortening is associated with inflammation and disease. At the end of the experiment, we found that the tumor-induction protocol decreased colon length non-signficantly in the control diet fed mice, and significantly in the HFD fed mice (**Figure 2B**). All mice given the tumor induction protocol developed visually identified polyps (100% incidence in both dietary AOM-DSS treatment groups), and no evidence of polyps was identified in the untreated animals. The HFD fed mice developed a greater multiplicity of tumors than mice fed the control diet (**Figure 2C**). We then collected stool and isolated lipids. Class specific scans were performed using triple quadrupole mass spectrometry (**Figure 2D**).

### Diet and tumors alter the bulk phospholipid quantities in stool

**Figure 3:**
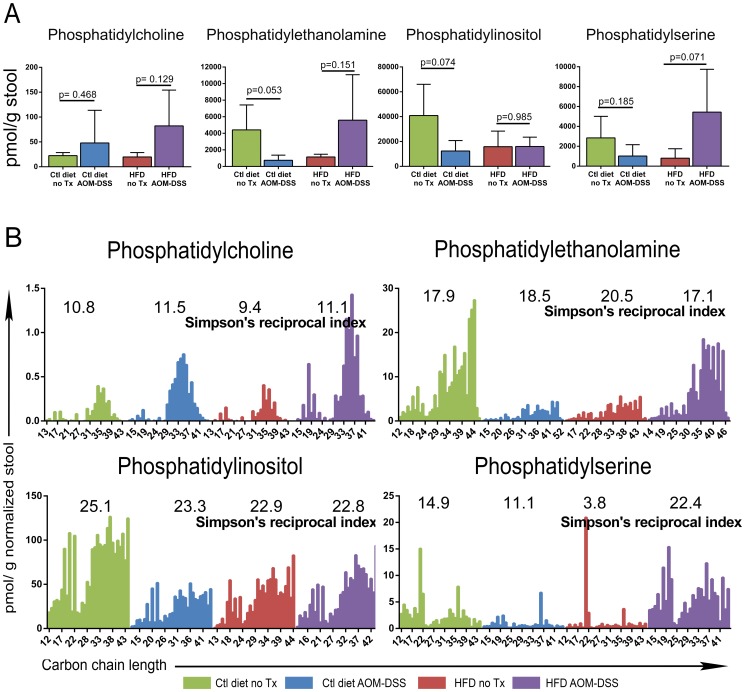
Quantities and distribution of lipid classes recovered from stool samples. Calculated pmol/g stool values for all detected peaks (both identified and unidentified) from representative class specific spectra were summed for each class of phospholipid. **A**) The bulk amount of each phospholipid class as determined above is graphed for each treatment group. Comparisons between untreated and AOM-DSS treated animals were analyzed by unpaired t-test. Bars represent mean ± SD, p values as indicated. **B**) The normalized pmol quantities of PLs were graphed. Chain lengths were summed and similar lengths combined to reduce complexity. Simpson's reciprocal diversity index for each treatment was calculated to determine α diversity. Bars represent means.

Mass spectrometric analysis revealed PI as the most abundant phospholipid and PC the least abundant phospholipid in the stool (**Figure 3A**). Tumor-bearing control diet fed mice (ctl diet AOM-DSS) tended to have decreased stool quantities of PI and PE when compared to untreated control diet fed mice (ctl diet no Tx), although this did not reach statistical significance. Tumor-bearing HFD fed mice (HFD AOM-DSS) had non-significantly increased quantities of several PLs (PC, PE, PS) compared to untreated HFD fed mice (HFD no Tx). These data demonstrate that both diet and inflammation associated tumors may lead to subtle changes in the stool quantities of PLs.

### Diversity of stool phospholipid carbon chain lengths is altered by diet and tumors

The diversity of the stool PL pool may be an important indicator of cell health and microbial community structure. Thus, we examined if a HFD or colitis-associated tumors altered the diversity of carbon chain lengths of the PL species identified by the LipidMaps database. As PLs have two carbon chains, the length of both chains was summed and the combined quantities of all similar length PLs plotted (**Figure 3B**). We examined the carbon chain length distribution for diversity using the Simpson's reciprocal diversity index. This calculation incorporates measures of both richness (the number of different lengths of chains) and evenness (the quantity of each chain length). The lowest possible value is 1, and greater values correspond to greater diversity. The diversity of PC, PE and PI was stable across the four treatment groups. However, the HFD reduced the diversity of PS compared to control diet fed mice (3.8 vs 14.9). Additionally, tumor-bearing control diet mice had less PS diversity than untreated control diet fed mice (11.1 vs 14.9). Finally, the tumor-bearing HFD fed mice had the greatest diversity of PS than any other group but especially compared to their untreated HFD fed counterparts (22.4 vs 3.8). These data highlight disease-specific changes in the diversity of PLs species.

### Clustering of phospholipid profile in untreated and tumor bearing mice

**Figure 4:**
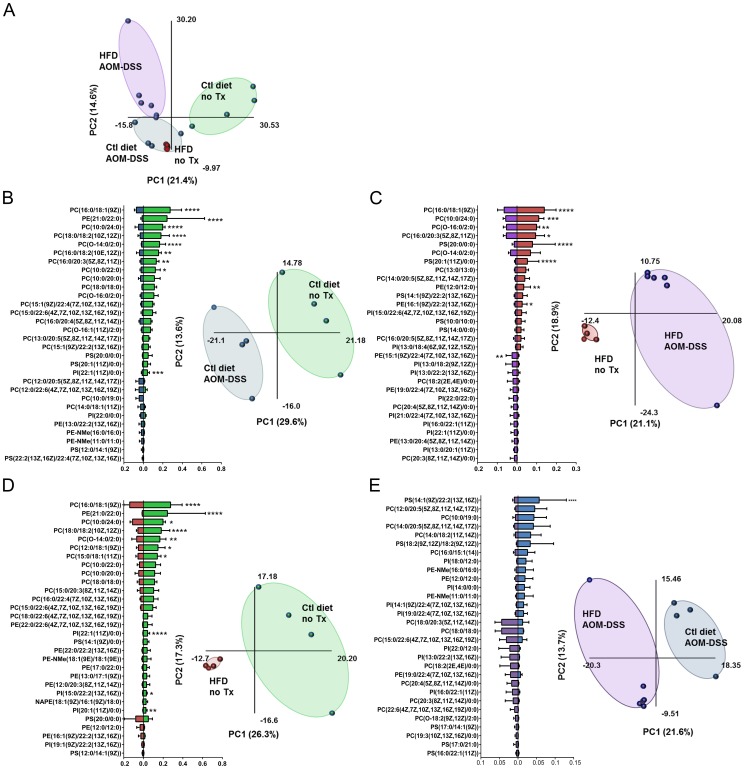
Distinct group clustering and signature expression profile of stool phospholipids. **A–E**) Partial least squares – discriminant analysis was performed on the PL profile of all the treatment groups combined (**A**) or in specific comparisons (**B–E**). Plots were generated using Multibase add-in for Excel. Bar graphs were generated using the relative pmol/g normalized stool weight quantities of the identified PL species from all four PL classes. **B–E**) Relative quantities of PLs were compared between treatment groups. The top 30 differential relative quantities between the treatment groups analyzed are presented. Bars represent mean ± SD. Each class of PL was analyzed separately. Significance determined by Two-way ANOVA, Bonferroni post-hoc test, **** = p<0.0001, *** = p<0.001, ** = p<0.01, * = p<0.05.

To determine if the PL profile could be used to differentiate between the different groups we performed a partial least squares – discriminant analysis (PLS-DA) of the PL profile. We chose to examine the relative abundance of the PL species rather than absolute magnitudes as we have already demonstrated bulk changes in PL class amounts (**Figure 3A**). Individuals within the four groups were closely clustered together, and demonstrated partial separation between groups. Interestingly, there was complete overlap of the PL profile between the untreated HFD fed mice and tumor-bearing control diet fed mice (**Figure 4A**), suggesting similarities between the untreated HFD fed stool PL profile and that of tumor bearing control diet fed mice.

### Differences in stool phospholipid profiles in tumor-bearing mice

In an effort to describe a dynamic signature of modulated PL species due to the presence of tumors we compared the relative abundance of PL species between untreated and tumor-bearing mice fed either the control (**Figure 4B**) or HFD (**Figure 4C**). Using relative PL quantities allows us to examine the movement of individual PL species in relation to the other PLs in its class. The top 30 PL species that varied between comparison groups were compiled to generate the signature of changes occurring between the treatment groups. Two thirds (20/30) of the most differentially abundant species in the comparison between untreated and tumor-bearing mice fed the control diet were PC species (**Figure 4B**). Partial least squares – discriminant analysis demonstrate clear clustering and separation of the PL profile between untreated and tumor-bearing control diet fed mice. When assessing the impact of tumors in mice fed a HFD, 11/30 of the differentially expressed PL species were also PC (**Figure 4C**). In both dietary conditions, PC species were most often significantly decreased in tumor-bearing mice. We found that regardless of diet, tumor-bearing mice had significant decreases in PC(16∶0/18∶1(9Z)), PC(10∶0/24∶0) and PC(16∶0/20∶3(5Z,8Z,11Z)) compared with their untreated dietary controls. Partial least squares – discriminant analysis between these two groups also demonstrated clear clustering and separation of the PL profile between untreated HFD and tumor-bearing HFD fed mice. These data demonstrate that there are PL patterns that are associated with tumors regardless of the host diet.

### Differences in stool phospholipid species based on diet

We then wished to know whether stool PLs were altered based on diet in otherwise healthy mice (**Figure 4D**). Of the differentially abundant PLs, the majority (13/30) were from the PC class. Similar to our finding that PC(16∶0/18∶1(9Z)) and PC(10∶0/24∶0) were significantly decreased in tumor-bearing stool, these two PCs were also significantly decreased in the untreated HFD fed mice compared to untreated control diet fed mice. The PLS-DA analysis identified differential clustering of the two groups based on their PL profile.

Only one PS species was significantly altered between tumor-bearing control diet fed and tumor-bearing HFD fed mice (**Figure 4E**). Similar to the other analysis, the main contributor to the list of the most differentially represented PLs were the PC class (14/30). The PLS-DA analysis demonstrated clustering of each treatment group and independence between the groups.

Overall, profiling of the PLs has demonstrated that tumors and a high fat diet decrease the relative quantity of several PC species when compared to healthy stool. However, fewer significant differences were observed between mice fed different diets when both were tumor-bearers (**Figure 4E**). This indicates that the profile of relative abundance of PLs may be used to differentiate between health and disease in certain settings.

### Tumor-bearing HFD fed mice have numerous species found infrequently in other groups

**Figure 5:**
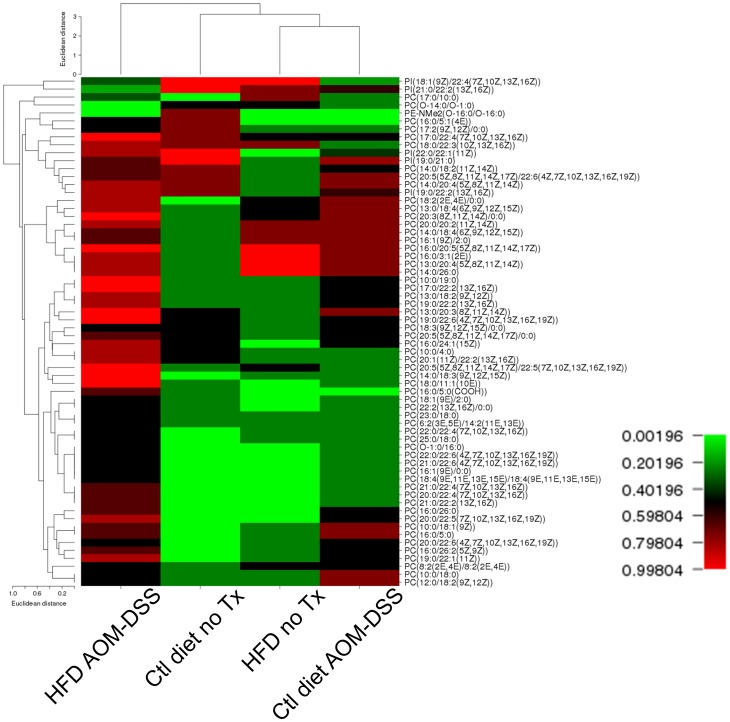
Low frequency species map. Phospholipid species that were present at low frequencies (less than 50%) in at least one of the four treatment groups were assembled into a list. Species that were not present in at least half of the samples in any one other group were removed. The frequency occurrence of each species was mapped by CIMminer. Phospholipid species and treatment groups were clustered by Euclidean distance.

We next examined low frequency PL species to determine if there were species that were over or under-represented in specific treatment groups. Species that were observed in fewer than half of the samples in a single group were compiled into a list and compared with the frequency with which they were found in all the other groups. Using this method, we generated a heat map hierarchy of the samples and clustered species and treatment groups (**Figure 5**). Euclidean clustering indicated that the tumor-bearing HFD fed mice were the most different from the other groups. Interestingly, the untreated HFD fed mice and the tumor-bearing control diet fed mice were clustered to each other rather than the untreated control diet fed group. The phospholipid class that was the primary contributor to this list was the PC class. Forty-four of the 52 low frequency species (85%) were PCs. These species were mainly present (greater than 50% frequency) in the stool of tumor-bearing HFD fed mice, but were found infrequently in the stool of the untreated mice. Tables outlining the distribution of low frequency species are included in **Table S1 & S2**.

To confirm that the changes in frequency of the lipid species were not due to the absence or presence of these species in the food, we examined the PL composition of the control diet and HFD. All the species that were assessed for low abundance in the stool were present in both the control and HFD food (data not shown).

### Stool microbiota is altered by diet and tumors

**Figure 6:**
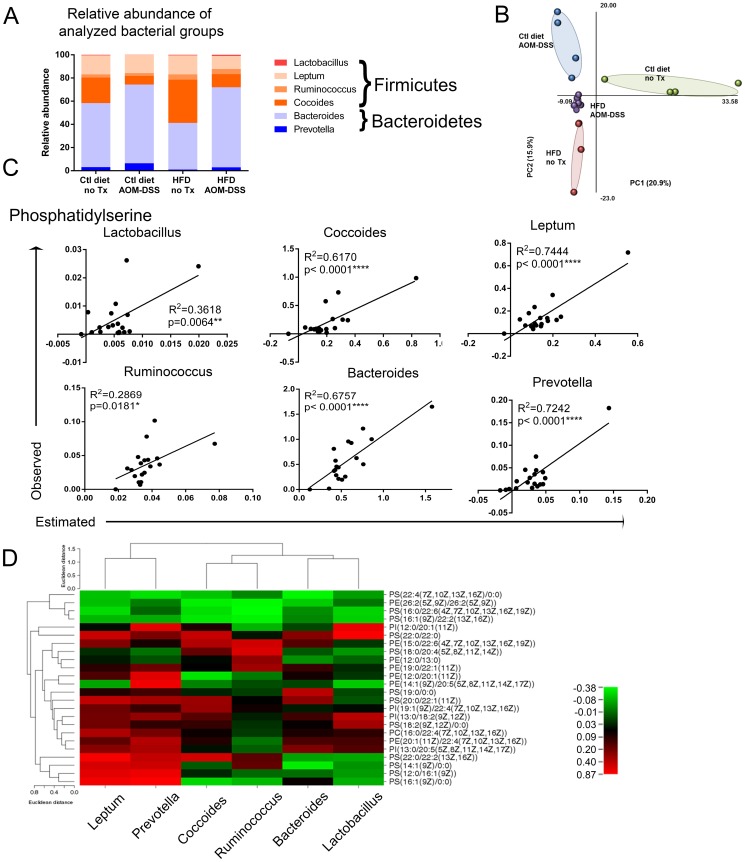
Correlations of relative bacterial abundance with PL levels. **A**) Relative quantities of bacterial groups were assessed in stool of treatment groups by qPCR. **B**) Partial least squares – discriminant analysis to determine grouping and clustering of samples was performed on ARISA data from stool samples. **C**) Partial-least squares regression was performed using the relative quantities of lipid species to predict the relative quantity of bacterial groups. The PS lipid class was the best predictor of bacterial group relative quantities. Significance of correlation between the observed and estimated values computed by partial least squares regression was determined by Pearson correlation. **D**) Significantly correlated PL species and bacterial groups (by Pearson correlation) were assembled into a list. The non-linear R^2^ value for the interaction between PL species quantities and bacterial quantities were then manually manipulated to reflect the direction of the interaction. These values were mapped by a one-matrix heat map (CIMminer). Phospholipids and bacterial groups were clustered by Euclidean distance.

Stool PLs are derived from host bile and shed epithelial cells but the microbiota is also a major contributor [35]. We examined the microbiota from parallel stool samples for the relative abundance of several individual bacterial groups (**Figure 6A** and **Figure S1A**) by qPCR analysis. The most abundant bacterial groups were the Gram-negative *Bacteroides* group and Gram-positive bacteria belonging to the *Clostridium leptum* group and the *Clostridium coccoides* group. We observed no significant changes in the total quantity of stool bacteria between the four treatment groups (**Figure S1A**). Consistent with previously published data [17], [36], the untreated HFD fed mice demonstrated a higher ratio of *Firmicutes* to *Bacteroidetes* (ratio 1.6) than control diet fed mice (ratio 0.6). Interestingly, after tumor induction in both the control diet and HFD fed mice the abundance of *Clostridium* coccoides group is reduced – although not significantly (**Figure S1A**).

We also generated bacterial composition profiles by ARISA and found distinct clustering patterns by PLS-DA of the different treatment groups (**Figure 6B** **and Figure S1B**). Comparisons based on the abundance of bacterial 16S-23S intergenic peaks/operational taxonomic units found complete separation of all four groups, indicating that the bacterial composition of each group was distinct (**Figure 6B**). Additionally, when we assessed the impact of a single factor – either diet or tumors – a clear separation between treatment groups was observed (**Figure S1B**).

### Prediction of bacterial group abundance by PL profile

We next aimed to model potential interactions between the stool PL profiles and bacterial group quantities. Using partial-least squares regression we are able to determine which bacterial group is most accurately predicted by the PL class quantities. The estimated values were plotted versus the observed values and Pearson correlations calculated to determine significance of the prediction. The best predictor of bacterial concentration was the PS class (**Figure 6C**). R^2^ values ranged from 0.28–0.74 and all plots were significant by Pearson correlation. The relative abundance of *Clostridium leptum* group and *Prevotella* group were most consistently estimated from the PS profile (R^2^ = 0.72–0.74) followed closely by *Bacteroides* (R^2^ = 0.67) and *Clostridium coccides* (R^2^ = 0.62). However, the relative abundance of *Lactobacilli/Enterococci* group and *Ruminococcus flavefaciens* subgroup were poorly estimated from the PS profile (R^2^ = 0.29–0.36). Partial least squares regression of PI and PE ineffectively estimated bacterial quantities (**Figure S2**).

Partial least squares regression plots were obtained by using the entire PL class profile to estimate the relative quantity of bacteria. However, we considered if individual PL species closely correlated to bacterial group quantities. Pearson correlations were applied to all PL species compared to bacterial group quantification (**Table 1**). Consistent with the partial least squares regression analysis each bacterial group was closely correlated with several members of the PS class. Fifteen of the 32 individual PL species that were significantly correlated to bacterial group quantities were PS (**Table 1**). Nine of the 32 PL species correlate with more than one bacterial group and 6 species correlate with more than two bacterial groups.

**Table 1:** Non-linear regression of bacterial group abundance(x) vs phospholipid abundance(y).

Name	*m/z*	Slope of the line	R^2^	Outliers (excluded, Q = 1.0%)	Number of XY Pairs	Pearson p value (two tailed)	P value summary
**Lactobacillus**							
PI(12∶0/20∶1(11Z))	808.64	1.330	0.644	1	16	0.0103	*
PI(13∶0/18∶2(9Z,12Z))	792.30	0.817	0.4003	1	15	0.0114	*
PS(18∶2(9Z,12Z)/0∶0)	521.34	0.345	0.3469	0	18	0.0101	*
**Coccoides**							
PS(22∶0/22∶2(13Z,16Z))	871.61	0.004	0.534	0	18	0.0006	***
PS(22∶0/22∶0)	903.77	0.002	0.4373	1	17	0.0038	**
PE(15∶0/22∶6(4Z,7Z,10Z,13Z,16Z,19Z))	749.55	0.033	0.3946	0	18	0.0052	**
PS(20∶0/22∶1(11Z))	873.72	0.003	0.326	1	17	0.0167	*
PI(19∶1(9Z)/22∶4(7Z,10Z,13Z,16Z))	926.93	0.010	0.3249	0	15	0.019	*
**Leptum**							
PS(22∶0/22∶2(13Z,16Z))	871.61	0.008	0.7208	0	18	<0.0001	****
PS(16∶1(9Z)/0∶0)	495.29	0.036	0.6707	0	18	<0.0001	****
PS(12∶0/16∶1(9Z))	677.45	0.025	0.5919	0	18	0.0002	***
PS(14∶1(9Z)/0∶0)	467.22	0.061	0.5415	1	17	0.0008	***
PS(22∶0/22∶0)	903.77	0.004	0.4442	1	17	0.0035	**
PS(20∶0/22∶1(11Z))	873.72	0.005	0.4262	1	17	0.0045	**
PC(16∶0/22∶4(7Z,10Z,13Z,16Z))	809.77	0.119	0.3841	1	16	0.0105	*
PI(13∶0/20∶5(5Z,8Z,11Z,14Z,17Z))	814.41	0.022	0.3062	1	15	0.0324	*
PE(15∶0/22∶6(4Z,7Z,10Z,13Z,16Z,19Z))	749.55	0.049	0.3006	0	18	0.0185	*
**Ruminococcus**							
PS(18∶0/20∶4(5Z,8Z,11Z,14Z))	811.53	0.113	0.5482	0	18	0.0004	***
PE(15∶0/22∶6(4Z,7Z,10Z,13Z,16Z,19Z))	749.55	0.394	0.468	0	18	0.0017	**
PS(16∶0/22∶6(4Z,7Z,10Z,13Z,16Z,19Z))	807.47	−0.044	0.3847	0	18	0.006	**
PE(26∶2(5Z,9Z)/26∶2(5Z,9Z))	963.73	−0.077	0.3668	1	17	0.0368	*
PE(19∶0/22∶1(11Z))	815.56	0.185	0.3316	1	17	0.0156	*
PE(12∶0/13∶0)	593.37	0.139	0.3174	0	18	0.0149	*
PS(16∶1(9Z)/22∶2(13Z,16Z))	813.53	−0.155	0.3048	0	18	0.0175	*
**Bacteroides**							
PS(19∶0/0∶0)	539.42	0.006	0.4058	1	17	0.006	**
PS(22∶4(7Z,10Z,13Z,16Z)/0∶0)	573.30	−0.006	0.3295	0	18	0.0127	*
PS(20∶0/22∶1(11Z))	873.72	0.002	0.3081	1	17	0.0207	*
PS(22∶0/22∶0)	903.77	0.001	0.3053	1	17	0.0215	*
**Prevotella**							
PS(16∶1(9Z)/0∶0)	495.29	0.156	0.8701	1	17	<0.0001	****
PE(14∶1(9Z)/20∶5(5Z,8Z,11Z,14Z,17Z))	707.46	0.152	0.6519	0	18	<0.0001	****
PS(12∶0/16∶1(9Z))	677.45	0.099	0.6467	0	18	<0.0001	****
PE(12∶0/20∶1(11Z))	689.58	0.091	0.5767	1	17	0.0004	***
PS(14∶1(9Z)/0∶0)	467.22	0.214	0.4591	1	17	0.0028	**
PS(22∶0/22∶2(13Z,16Z))	871.61	0.023	0.4343	0	18	0.0029	**
PE(20∶1(11Z)/22∶4(7Z,10Z,13Z,16Z))	821.45	0.105	0.3998	1	17	0.0065	**
PI(13∶0/20∶5(5Z,8Z,11Z,14Z,17Z))	814.41	0.090	0.3696	1	15	0.0162	*
PS(20∶0/22∶1(11Z))	873.72	0.018	0.369	1	17	0.0097	**
PI(13∶0/18∶2(9Z,12Z))	792.30	0.111	0.3232	1	15	0.027	*

Phospholipid and bacterial group abundance was calculated as relative abundance. Non-linear regression calculated with a straight line equation. Outlier determination automatically computed with Q = 1%. Pearson correlations were calculated for each comparison.

Finally, the significant correlation values (non-linear R^2^ value corrected to positive or negative depending on the slope of the line) between individual PL species and at least one bacterial group was plotted in a heat map (**Figure 6D**). Euclidean clustering analysis demonstrates that based on correlation strength to specific PL species the bacterial groups cluster in three distinct groups. *Clostridium leptum* and *Prevotella* share both positive and negative correlations with a number of PL species. *Clostridium coccoides* and *Ruminococcus flavefaciens* cluster together with similar correlations to PL species and finally the *Bacteroides* and *Lactobacilli/Enterococci* groups clustered with similar PL correlations. The observed clustering of the bacterial groups based on PL correlations is in contrast to their taxonomic clustering based on 16S rDNA gene sequence. Our results may indicate that PL usage between closely genetically related bacterial species may be distinct. Further studies examining the PL profile of closely related intestinal-derived bacterial species will be needed to address this question.

## Discussion

Both a HFD [1] and obesity increase colorectal cancer (CRC) risk and obesity confers a worse outcome [1]–[3]. The microbiota is altered by diet [16], [17] but the consequence of these changes to both host-derived and microbiota-derived metabolites is currently unknown. In this exploratory assessment, we investigated stool phospholipids—an understudied class in metabonomics, and the changes induced by diet or colitis-associated tumors and their relationship to changes in the microbiota.

We show that the PL profile of stool is altered both by diet and tumors and may be useful as a biomarker for disease screening or may be targeted for therapeutic intervention. A HFD increases weight gain and tumor formation in mice. We have shown that both diet and tumors alter the stool PL profile and that this is both dependent and independent of the changes that occur to the microbiota. We have generated PL profiles based on diet and tumors and identified species that occur frequently in tumor-bearing mice fed a HFD that are not present in other groups. Collectively, our observations indicate that stool is an excellent source to examine both host-derived and microbiota-derived changes of the PL profile that may reflect disease pathogenesis.

The metabolome has been studied in both inflammatory bowel disease (IBD) and colon cancer with several classes of metabolites consistently regulated in each disease state. In IBD, several studies have examined metabolites in serum and stool by ^1^HNMR and found increased quantities of amino acids [37]–[39] and decreased quantities of short-chain fatty acids (SCFA) including butyrate and acetate and decreased methylamine and trimethylamine [37]. Metabolites that are increased in ulcerative colitis (UC) patient's feces were taurine and cadaverine and bile acids [40], [41]. Choline and its derivatives tri-methlamine *N*-oxide (TMAO) and betaine were increased in both IBD [42] and cardiovascular disease [43]. In colorectal cancer, urine and tissue amino acids were elevated as well as urea cycle metabolites in both mouse and human tissues [44]. Finally, diet alone has also been shown to alter metabolic parameters in as few as 2 weeks [45].

We observed no increase in the total quantity of phospholipids in response to feeding a HFD in otherwise untreated mice (**Figure 3A**). While several previous studies have observed an increase in stool lipids following HFD feeding [46], [47] these reports described the levels of total free fatty acids, which primarily reflects the degree of triglyceride absorption in the small intestine. By contrast, we have focused on PLs, which largely reflect endogenous PL levels including those generated by the microbiota.

Phosphatidylinositiol was the most abundant PL of the analyzed classes. Colitis-associated tumors in mice fed a control diet led to non-significant decreases in bulk PI and PE quantities in the stool compared to untreated controls. The importance of PIs has recently been identified in several inflammatory models. Dietary addition of PIs prevented Concanavalin A-induced inflammation in a model of hepatitis [48] and was shown to modulate weight gain in a diet-induced obesity model [49]. Additionally, PI derivatives have been shown to modulate immune activation and function in T cells. Intraperitoneal injection of PI during 2,4,6-trinitrobenzene sulfonic acid (TNBS) induced colitis decreased T cell inflammatory cytokine release and improved histological tissue scoring [50]. As exogenous addition of PI in the previous studies ameliorated inflammation, our results indicating reduced stool quantities of PI in colitis-associated tumor bearing mice are consistent with a pro-inflammatory state. Future studies examining the protective effect of PIs on tumor induction in this model would be of interest. Inositol has been found to decrease the frequency of tumors in a model of colitis-associated neoplasia [51] and is currently being tested as an intervention in UC patients with low-grade dysplasia (Clinicaltrials.gov identifier: NCT01111292). Unfortunately, the mass spectrometric analysis that we performed cannot distinguish the stereoisomerism of the identified PI species, thus we cannot infer their signaling function.

We next assessed the diversity of the PLs based on their overall carbon side chain length (**Figure 3C**). Assessments of diversity in both macro and micro-ecological environments are measures of the stability and robustness of the environment. Our analysis indicates that feeding a HFD greatly reduces the diversity of PS species compared to untreated mice fed the control diet. Using PLs as a surrogate marker for micro-environmental health may allow for a composite analysis of the health of both the host and the microbiota as stool PLs are composed of both host-derived and microbiota-derived species [35].

The PL profiles suggest that healthy mice – fed either a control or a HFD, have several significantly over-represented PC species compared to their tumor-bearing counterparts. PCs are not reported to be a dominant class of PLs in bacteria, although they have been observed as a minor constituent of several bacterial species [52], [53]. In contrast, PCs are the dominant PL class in mucus [54], [55]. Several PC species were decreased in HFD (**Figure 4D**) and colitis-associated tumor bearing mice (**Figure 4B**) compared to untreated control diet fed mice. It is possible that loss of these PC species may alter the mucus layer reducing its hydrophobicity allowing bacterial binding to the epithelium and exacerbating inflammation leading to tumor promotion. Disruption to the mucus layer has been investigated as a potential therapeutic target in ulcerative colitis and two studies have suggested amelioration of UC following colonic delivery of a mixture of PLs containing 30% PC [56], [57].

The microbiota is a major contributor to the stool PL profile and is an important determinant to overall health. Diet modifies the microbiota. Several studies have demonstrated that obesity is associated with lower proportions of Bacteroidetes and higher proportions of Firmicutes in both human and murine models [16], [18], [58]. Turnbaugh *et al*. have shown that transfer of the microbiota from genetically obese (*ob/ob*) [18] and diet-induced obese mice [59] to lean animals increased fat mass and energy extraction in the recipient animals. Importantly, studies have also shown that the composition of microbiota can be altered by changes to the diet within a short period of time [58]. Our study has demonstrated differences in the microbiota profile induced by diet and tumors.The microbiota has an important role in disease, and is altered in both IBD and in cancer. The microbiota of Crohn's disease patients has reduced species diversity [60]–[62], and UC patients have increased bacterial density in close association with the mucosa [63]. Studies investigating the relationship between the microbiota and colon cancer have shown decreases in protective butyrate-producing bacterial species and increases in harmful hydrogen sulfide producing species [64]. The conversion of bile acids by the microbiota to carcinogenic secondary bile acids [65] has also been hypothesized to contribute to colon cancer development.

A recent study by McHardy *et al* has examined the metabolic profile of colonic washes in human subjects with microbial populations and found significant correlations between them [66]. In our study, partial least squares regression demonstrated that the relative quantities of stool PS species had the greatest predictive power for several of the bacterial groups, but not all of them (**Figure 6C**). This may be attributable to the characteristic PL usage by the different bacterial groups, but it will require systematic analysis of the PL profile of isolated bacterial strains to verify this hypothesis.

Relative quantities of individual PL species also correlated to the bacterial group quantities. PL species were both positively and negatively correlated with bacterial group quantities. The individual species correlations were mostly between PS and PE species and the bacterial groups. This is consistent with reports of the predominant PL species in bacteria [67] and provides preliminary evidence that PL profiling may be useful for quantifying and identifying the microbiota. Interestingly, the clustering of bacterial groups with PL species is not consistent with their 16S rDNA phylogenic distances. The *Clostridium leptum* and *coccoides* bacterial groups were not grouped together based on their correlations to PL species and it is possible that despite being genetically related, these two bacterial groups may be diverse in their PL usage. The PL profile of different bacterial species is currently an under-studied field, especially since culturing individual bacterial species from the intestinal microbiota is technically difficult. This will be an area of important future study as determining the PL profile of closely related bacterial groups may elucidate differences in the organization of bacterial plasma membranes which may exist even within the same genus. Deciphering the PL profile of bacteria may provide novel functional bacterial classifications or provide useful targets for modulating the microbiota.

## Conclusions

Phospholipids are the building blocks of cells of both the host and the microbiota and as such may be important indicators of health. Phospholipids are altered in disease and profiling them may be of predictive value in pathological states. We have described that PLs isolated from stool have a characteristic profile based on diet and tumor status and that certain PL classes can be correlated to particular bacterial groups. The emergence of more affordable technologies to sequence the metagenome will undoubtedly allow for more detailed study of the impact of changes in the proportion of microbial genes for PL synthesis and metabolism on the stool PL profile. Advances in understanding the function of individual PL species are also ongoing and there is a great potential for lipidomics to inform a number of disease processes.

## Supporting Information

Figure S1 **Quantification of stool bacterial groups.** **A**) Quantities of bacterial groups were determined by qPCR. **B**) Partial least squares- discriminant analysis of comparisons of interest based on ARISA bacterial distributions.
(TIF)

Figure S2 **Partial-least squares regression was performed using the relative quantities of lipid species to predict the relative quantity of bacterial groups.** Significance of correlation between the observed and estimated values computed by partial least squares analysis was determined by Pearson correlation.
(TIF)

Table S1 **Frequency of low abundance species in each group.**
(PDF)

Table S2 **Low frequency species in a single group.**
(PDF)

Table S3 **Raw phospholipid data file.**
(XLSX)

Dataset S1 **Normalized phospholipid and bacterial quantities.**
(XLSX)
