# Supplementary figures and images for: Stool Phospholipid Signature is Altered by Diet and Tumors
Source: PLoS One. 2014 Dec 3;9(12):e114352. doi: 10.1371/journal.pone.0114352 (PMC4254978; doi:10.1371/journal.pone.0114352)

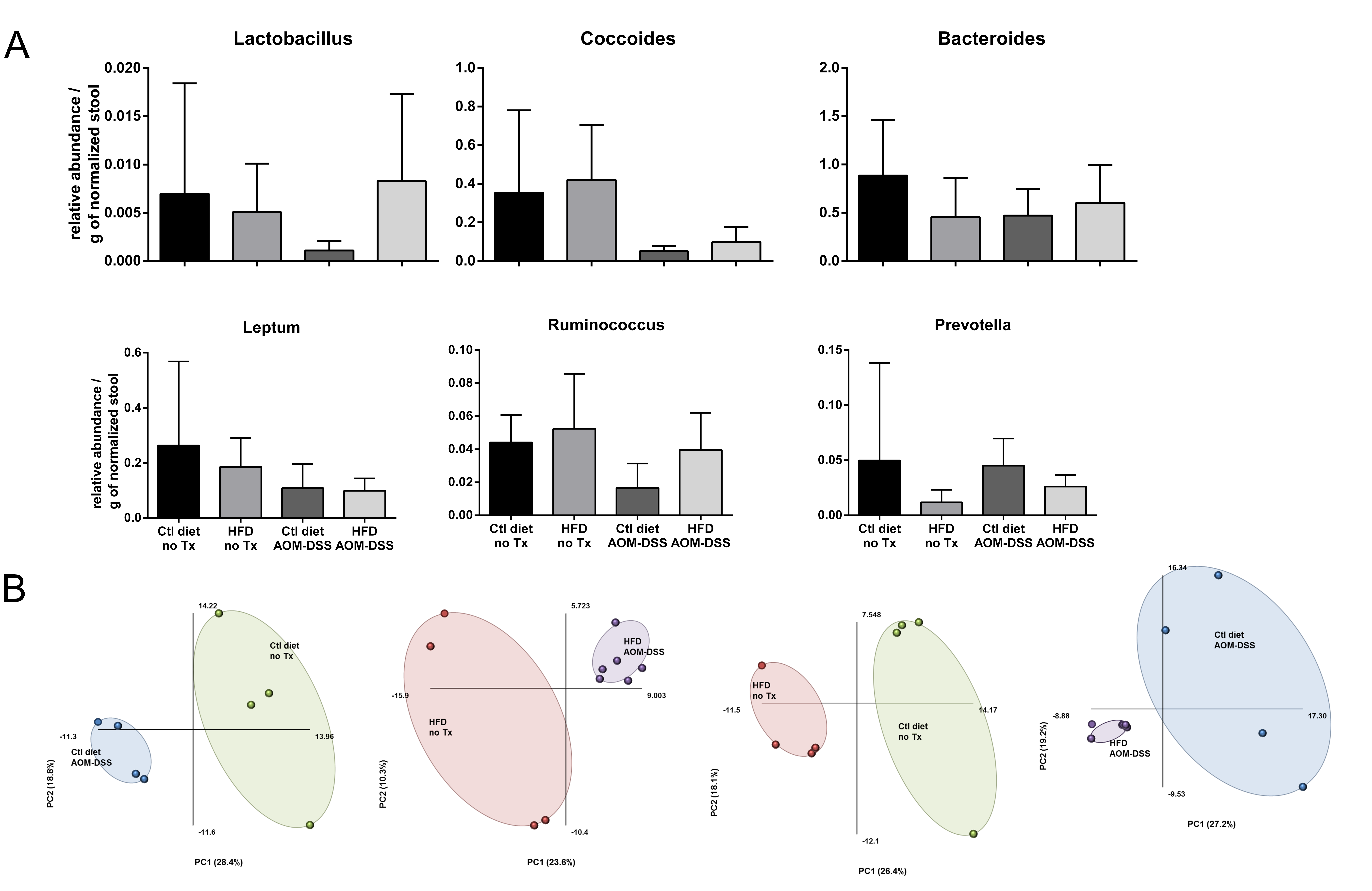

Supplement: Figure S1 — Quantification of stool bacterial groups. A) Quantities of bacterial groups were determined by qPCR. B) Partial least squares- discriminant analysis of comparisons of interest based on ARISA bacterial distributions. (TIF) [file pone.0114352.s001.tif]
