# Supplementary material for: Stool Phospholipid Signature is Altered by Diet and Tumors
Source: PLoS One. 2014 Dec 3;9(12):e114352. doi: 10.1371/journal.pone.0114352 (PMC4254978; doi:10.1371/journal.pone.0114352)
Supplement: Table S1 — Frequency of low abundance species in each group. (PDF) [file pone.0114352.s003.pdf]

Table S1 . Frequency of low abundance species in each group

Name	*m/z*	Ctl diet no Tx	HFD no Tx	Ctl diet AOM-DSS	HFD AOM-DSS
**Species found primarily in healthy control diet fed mice**					
PE-NMe2(O-16:0/O-16:0)	678.54	3/4	0/4	0/4	0/4
**Species found primarily in healthy HFD fed mice**					
PC(17:0/10:0)	663.85	0/4	3/4	1/4	2/6
**Species found primarily in tumor bearing HFD fed mice**					
PC(10:0/19:0)	691.87	1/4	1/4	2/4	6/6
PC(13:0/18:2(9Z,12Z))	715.78	1/4	1/4	2/4	5/6
PC(14:0/18:3(9Z,12Z,15Z))	727.21	0/4	1/4	1/4	6/6
PC(16:0/26:0)	873.68	0/4	0/4	2/4	4/6
PC(16:0/26:2(5Z,9Z))	869.62	0/4	1/4	2/4	4/6
PC(16:0/5:0(COOH))	609.07	1/4	0/4	0/4	4/6
PC(17:0/22:2(13Z,16Z))	827.24	1/4	1/4	2/4	6/6
PC(18:0/11:1(10E))	689.87	1/4	0/4	1/4	6/6
PC(19:0/22:1(11Z))	857.66	0/4	1/4	2/4	5/6
PC(19:0/22:2(13Z,16Z))	855.72	1/4	1/4	2/4	5/6
PC(20:0/22:4(7Z,10Z,13Z,16Z))	865.85	0/4	0/4	1/4	4/6
PC(20:0/22:5(7Z,10Z,13Z,16Z,19Z))	863.39	0/4	0/4	2/4	5/6
PC(21:0/22:2(13Z,16Z))	883.40	0/4	0/4	1/4	4/6
PC(21:0/22:4(7Z,10Z,13Z,16Z))	879.36	0/4	0/4	1/4	4/6
**Species found primarily in healthy mice (both control diet and HFD)**					
PI(18:1(9Z)/22:4(7Z,10Z,13Z,16Z))	912.74	4/4	4/4	1/4	2/6
**Species found primarily in tumor bearing mice (both control diet and HFD)**					
PC(10:0/18:1(9Z))	677.32	0/4	1/4	2/4	4/6
PC(16:0/5:0)	579.68	0/4	1/4	3/4	4/6
**Species found primarily in control diet fed mice (both healthy and tumor-bearing)**					
none					
**Species found primarily in HFD fed mice (both healthy and tumor-bearing)**					
none					
