# Supplementary material for: Stool Phospholipid Signature is Altered by Diet and Tumors
Source: PLoS One. 2014 Dec 3;9(12):e114352. doi: 10.1371/journal.pone.0114352 (PMC4254978; doi:10.1371/journal.pone.0114352)
Supplement: Table S2 — Low frequency species in a single group. (PDF) [file pone.0114352.s004.pdf]

Table S2. Low frequency species in a single group

Name	*m/z*	Ctl diet no Tx	HFD no Tx	Ctl diet AOM-DSS	HFD AOM-DSS
**Species not found in healthy control diet fed mice**					
PC(14:0/18:4(6Z,9Z,12Z,15Z))	725.59	1/4	3/4	3/4	4/6
PC(13:0/20:4(5Z,8Z,11Z,14Z))	739.51	1/4	4/4	3/4	5/6
PC(14:0/26:0)	845.96	1/4	4/4	3/4	5/6
PC(20:0/20:2(11Z,14Z))	841.62	1/4	3/4	3/4	5/6
PC(16:0/20:5(5Z,8Z,11Z,14Z,17Z))	779.53	1/4	4/4	3/4	6/6
PC(16:1(9Z)/2:0)	535.44	1/4	3/4	3/4	4/6
PC(20:3(8Z,11Z,14Z)/0:0)	545.36	1/4	2/4	3/4	6/6
**Species not found in healthy HFD fed mice**					
PC(13:0/20:3(8Z,11Z,14Z))	741.70	2/4	1/4	3/4	6/6
PC(14:0/20:4(5Z,8Z,11Z,14Z))	753.77	3/4	1/4	3/4	5/6
PI(19:0/21:0)	922.50	4/4	1/4	3/4	4/6
PI(19:0/22:2(13Z,16Z))	932.69	3/4	1/4	3/4	5/6
**Species not found in tumor bearing control diet fed mice**					
PC(18:0/22:3(10Z,13Z,16Z))	839.62	3/4	3/4	1/4	5/6
**Species not found in tumor bearing HFD fed mice**					
PI(21:0/22:2(13Z,16Z))	960.68	4/4	3/4	3/4	1/6
PI(14:1(9Z)/22:4(7Z,10Z,13Z,16Z))	856.46	3/4	3/4	2/4	2/6
PI(20:2(11Z,14Z)/0:0)	624.44	4/4	4/4	4/4	2/6
